# Preventive hyperbaric oxygen therapy improves acute graft-versus-host disease by activating the Nrf2/HO-1 pathway

**DOI:** 10.3389/fimmu.2025.1529176

**Published:** 2025-02-27

**Authors:** Chao Xue, Hao Chen, Yiou Zhao, Dai Yuan, Xiaosheng Fang, Mei Ding, Huiting Qu, Xin Wang, Xueling Ge, Kang Lu, Yujie Jiang

**Affiliations:** ^1^ Department of Hematology, Shandong Provincial Hospital, Cheeloo College of Medicine, Shandong University, Jinan, China; ^2^ Department of Hematology, Shandong Provincial Hospital Affiliated to Shandong First Medical University, Jinan, China; ^3^ Department of Hematology, Peking University First Hospital, Beijing, China; ^4^ Department of Hyperbaric Oxygen Medicine, Shandong Provincial Hospital Affiliated to Shandong First Medical University, Jinan, China; ^5^ College of Life Science and Technology, Changchun University of Science and Technology, Changchun, China; ^6^ School of Medicine, Shandong University, Jinan, Shandong, China

**Keywords:** allogeneic hematopoietic stem cell transplantation, acute graft-versus host disease, hyperbaric oxygen therapy, erythroid-derived 2-related factor 2 (Nrf2), reactive oxygen species

## Abstract

**Background:**

Hyperbaric oxygen therapy (HBOT) has been confirmed as an effective and economical therapeutic modality for treating hemorrhagic cystitis (HC), whether induced by infection or acute graft-versus-host disease (aGVHD), in transplant recipients. However, its potential benefits in treating aGVHD remain largely unknown. This study explored the effects of HBOT on aGVHD and its underlying mechanisms.

**Methods:**

The beneficial effects of HBOT on aGVHD were investigated in a murine model. Manifestations, pathological alterations, reactive oxygen species (ROS) levels in target organs, and survival data of the recipient mice were collected. Nuclear factor erythroid-derived 2-related factor 2 (Nrf2) and its downstream enzyme heme-oxygenase 1 (HO-1) expression in mouse samples were assessed via Western blot and immunohistochemistry analyses. ML385, an Nrf2 inhibitor, was used to validate the protective role of Nrf2 in the beneficial effect of HBOT on aGVHD. Furthermore, we initiated a clinical cohort study and collected data from the patients with definite aGVHD before and after HBOT to validate the preclinical conclusions.

**Results:**

We found that HBOT alleviated aGVHD in mice, which was associated with a significantly prolonged overall survival (OS) and reduced pathological injury, whereas Nrf2 inhibition had the opposite effect. HBOT decreased ROS levels and proinflammatory cytokines, including IL-6 and TNF-α, while upregulated Nrf2 and its downstream antioxidant enzyme HO-1. In the clinical cohort study, the incidence of grades 1–3 aGVHD was significantly lower in the combination arm containing HBOT than in the HBOT-free cohort.

**Conclusion:**

Preventive HBOT can mitigate aGVHD by activating the Nrf2/HO-1 signal transduction pathway, suggesting that HBOT may be a feasible approach for both the prevention and treatment of aGVHD.

**Clinical trial registration:**

ClinicalTrials.gov, identifier NCT04502628.

## 1 Introduction

Allogeneic hematopoietic stem cell transplantation (allo-HSCT) is a potentially curative therapeutic strategy for patients with hematopoietic malignancies. However, its therapeutic benefits and broader application are limited by acute graft-versus-host disease (aGVHD), which remains a major obstacle to long-term survival in this population. Glucocorticoids and multiple immunosuppressive agents, including calcineurin inhibitors (CNIs), mycophenolate mofetil (MMF), anti-CD25 antibodies, and JAK1/JAK2 inhibitors, are often used alone or in combination to treat aGVHD in clinical scenarios. Despite these treatments, 30%–50% of patients develop steroid-refractory/resistant aGVHD (SR-aGVHD), and more intensive immunosuppressive therapies are associated with an increased risk of infection and malignancy relapse (1, 2). Therefore, novel aGVHD prophylactic and therapeutic strategies that offer superior efficacy, safety, and cost-effectiveness while being less technically demanding remain urgently needed.

Reactive oxygen species (ROS) are primary triggers of the inflammatory response and play a key role in the pathogenesis of aGVHD (3–8). Therefore, strategies targeting ROS production may be effective for managing aGVHD. Nuclear factor erythroid-derived 2-related factor 2 (NFE2L2, or Nrf2) serves as a master regulator of cellular redox balance, detoxification, and stress pathways. Under normal conditions, Nrf2 remains in the cytoplasm bound to Kelch-like ECH-associated protein 1 (Keap 1) but translocates to the nucleus after ROS stimulus (9). Nrf2 activation upregulates downstream antioxidant enzymes, such as heme-oxygenase 1 (HO-1) and superoxide dismutase (SOD). These findings suggest that activating Nrf2 may inhibit oxygen-free radicals and protect host organs from injury during aGVHD.

Hyperbaric oxygen therapy (HBOT) is a well-established treatment method used to improve nonhealing ulcers secondary to aGVHD and hemorrhagic cystitis (HC) after allo-HSCT, whether induced by infection or aGVHD (10, 11). Although the exact mechanism is not fully understood, HBOT has been demonstrated to reduce the release of various proinflammatory cytokines and enhance the efficacy of antibiotics (12, 13). Previous studies have documented the positive involvement of Nrf2 during HBOT with traumatic brain injury and diabetic foot ulcers (14, 15). In this study, we demonstrated that the protective effects of HBOT were associated with the activation of the Nrf2/HO-1 signaling pathway in an allo-HSCT aGVHD mouse model. These experimental findings were further verified in 83 patients who developed aGVHD after allo-HSCT.

## 2 Materials and methods

### 2.1 Mice

Specific pathogen-free (SPF) male C57BL/6 (H-2K^b^) mice (5–6 weeks old, 18–22 g) and female BALB/C (H-2K^d^) mice (7–8 weeks old, 18–22 g) were purchased from the Laboratory Animal Center of Shandong University (Shandong, China) and housed under SPF conditions. All animal protocols were approved by the Animal Care and Use Committee of Shandong University (Permit Number: 2018-0004) and complied with the Guide for the Care and Use of Laboratory Animals published by the National Institutes of Health (NIH Publication, Eighth Edition, 2011). The animals were killed upon reaching the humane endpoint established by the Animal Ethics Committee (30% weight loss or signs of moribundity). This study adhered to the ARRIVE guidelines (https://www.nc3rs.org.uk/arrive-guidelines).

### 2.2 Patients and clinical study design

Between September 2020 and January 2022, 83 potentially eligible aGVHD patients (aged 18–60 years) with hematological diseases who underwent allo-HSCT in the Department of Shandong Provincial Hospital were prospectively identified and enrolled in the present study. All participants were randomly assigned to either the standard cohort or the HBOT cohort at a 2:1 ratio through simple randomization without stratification. CNIs combined with MMF, along with short-term methotrexate, were used as the standard prevention regimen for aGVHD. If aGVHD occurred, 1–2 mg/kg of prednisone (or an equivalent dose of methylprednisolone) was added to the treatment. In the experimental group, in addition to this treatment, HBOT was administered as part of the protocol. The HBOT cohort was treated in an enclosed HBOT chamber with 100% oxygen for 90 min at an absolute pressure of 202.6 kPa (equivalent to 2 atmospheres [ATM]), starting at the first occurrence of aGVHD. The treatments lasted for 20 days, and the therapeutic effect of HBOT was assessed using the aGVHD Glucksberg grading system (16). The exclusion criteria included a medical history of claustrophobia, poor physical condition, or unstable vital signs. Participants could be withdrawn at any time due to discomfort or other side effects, such as tinnitus. All data were analyzed anonymously to protect patient privacy, and the protocols and consent forms were approved by the Human Subjects Review Committee of Shandong Provincial Hospital. The prospective study adhered to the Declaration of Helsinki and was registered at https://clinicaltrials.gov/ct2/show/NCT04502628.

### 2.3 aGVHD animal model

The hematopoietic cell transplantation procedure was performed as previously described (17, 18). Recipient BALB/C mice underwent lethal total body irradiation at a dose of 750 cGy (total dose), adjusted based on weight. Within 4–6 h of irradiation, the recipients were reconstituted via intravenous injection of either bone marrow cells (1 × 10^7^ cells) alone (BM) or bone marrow cells combined with purified spleen cells (2 × 10^7^ cells) (aGVHD) from C57BL/6 donor mice (19). Sham mice, serving as a total body irradiation (TBI) control, received the same volume of phosphate-buffered saline (PBS) via the tail vein. Recipient mice were monitored daily for survival and scored weekly for clinical aGVHD, including posture, activity, fur condition, skin integrity, and weight loss, as described previously (19). Mice were either observed or killed for histopathological and flow cytometric analyses. The severity of GVHD was evaluated using a five-criteria scoring system from Cooke et al., which includes weight loss (1: 10%–25%; 2: > 25%), posture (1: mild hunching only at rest; 2: severe hunching impairs movement), mobility (1: stationary for > 45% of the time; 2: stationary unless stimulated), fur texture (1: mild to moderate ruffling; 2: ruffling over the entire body), and skin integrity (1: scaling paws/tails; 2: significant areas of denuded skin).

### 2.4 HBOT in the aGVHD mouse model

HBOT was administered to recipient mice once daily starting, on Day 7 posttransplantation, and continued for an additional 2 weeks (aGVHD+HBOT and BMT+HBOT groups). The aGVHD group did not received any treatment after transplantation for comparison, while the BMT+HBOT group served as a control for HBOT toxicity. HBOT was delivered in a sealed chamber at a pressure of 2.4 atmosphere absolute (ATA) for 90 min per day. Clinical symptoms of aGVHD were monitored and recorded daily. The severity of aGVHD was assessed using a composite aGVHD scoring system (Cooke et al., 1996) every 7 days (19). Recipient mice were killed on Day 28 posttransplantation, and target organs (liver, colon, small intestine, skin, lung, and spleen) were harvested for histopathological, Western blot, immunohistochemistry, and immunofluorescence analyses.

An Nrf2 inhibitor (ML385, Selleck, Shanghai, China) was used to pretreat recipient mice in the aGVHD-HBOT group (aGVHD-HBOT-ML385 group). ML385 was administered 2 h before HBOT via oral gavage at 20 mg/kg/day in a volume of 200 μL per dose per mouse. Vehicle and ML385 dosing began on Day 7 posttransplantation (day +7) and continued daily for 2 weeks. No acute toxicity of ML385 was observed in the animal experiments.

### 2.5 Flow cytometry analysis

Recipient mice were killed 10 days post-HSCT, and BM cells were harvested to determine the chimeric status. Standard flow cytometric surface staining protocols were used as previously described (20). Briefly, BM cells were labeled with a mouse fluorescein isothiocyanate (FITC)-conjugated anti-H-2K^b^ antibody and an allophycocyanin (APC)-conjugated anti-H-2K^d^ antibody. Flow cytometry was performed using a FACS auto flow cytometer (BD Biosciences, San Jose, CA, USA), and data analysis was performed with FlowJo software (FlowJo, LLC, Oregon, USA).

### 2.6 Histopathological analysis

To examine the histopathological alterations, mice from each cohort were killed by cervical dislocation on Day 28 post-HSCT. The liver, colon, small intestine, skin, lung, and spleen were collected. All the tissue samples were fixed in 4% paraformaldehyde for 24 h and then embedded in paraffin. The tissue sections were routinely stained with hematoxylin and eosin (H&E) and evaluated in a blinded fashion under a light microscope. The histological assessment of aGVHD target organs was performed as described previously (21, 22). Lung injury was assessed semiquantitatively based on several parameters, including alveolar septal thickening, hemorrhage, inflammatory cell infiltration, and consolidation. Liver injury was evaluated based on bile duct damage and infiltration of inflammatory cells. Gut GVHD was scored based on crypt apoptosis and lamina propria inflammation. Skin GVHD was scored based on the tissue damage in the epidermis and dermis, as well as the loss of subcutaneous fat. The scoring system for each parameter, which evaluated both the extent and severity of tissue damage, used the following scale: 0, normal; 0.5, focal and rare; 1, focal and mild; 2, diffuse and mild; 3, diffuse and moderate; and 4, diffuse and severe. The scores for each parameter were then summed to provide a total score for each sample.

### 2.7 Immunofluorescence analysis of ROS detection

The levels of ROS in the targeted tissues were measured using a reactive oxygen species assay kit according to the manufacturer’s instructions (Sigma-Aldrich, St. Louis, MO, USA). For immunofluorescence staining, an optimal cutting temperature (OCT) compound (Sakura Finetek, Tokyo, Japan) was used to prepare frozen sections of fresh tissue samples. The fluorescence images were captured and analyzed with a Leica DMi8 confocal microscope (Leica, Wetzlar, Germany).

### 2.8 Cytokine analysis by Luminex technology

Terminal blood samples were collected from the inner canthus venous plexus of recipients 28 days after HSCT. Multiplex quantification of cytokines (interleukin [IL]-2, IL-4, IL-6, IL-10, IL-17, tumor necrosis factor-alpha [TNF-α], interferon gamma [IFN-γ], and HGF) in the serum was performed via the 8-Plex Mouse Magnetic Luminex Assay (R&D Systems, Minneapolis, MN, USA) according to the manufacturer’s directions. Using a Luminex-200 instrument, the samples were analyzed as single replicates, and the standards were analyzed as duplicates.

### 2.9 Quantitative reverse transcription-polymerase chain reaction analysis

The mice in the different groups were euthanized, and liver tissues were removed. Total RNA was extracted from aGVHD target organs with TRIzol reagent (Takara, Tokyo, Japan) according to the manufacturer’s instructions. The transcription levels of the Nrf2 and HO-1 genes were analyzed by real-time PCR using SYBR Green Master Mix (Toyobo, Osaka, Japan). The relative quantification of the target gene was performed using the β-actin housekeeping gene, following the 2^−ΔΔCt^ method.

### 2.10 Immunohistochemistry

Formalin-fixed, paraffin-embedded mouse tissue sections were used for immunohistochemistry, and the experiments were performed following standard procedures. All histological analyses were performed in a blinded fashion for the experimental groups.

### 2.11 Western blot analysis

For immunoblotting detection of target proteins, mouse liver tissue extracts were prepared as described previously (23). The supernatants were stripped and incubated with an anti-β-actin antibody to determine the amount of protein present in each lane. The protein bands were detected with an enhanced chemiluminescence agent and quantified with ImageJ software.

### 2.12 Statistical analysis

Graphing and statistical analysis were performed with GraphPad Prism 8.0 software (GraphPad Software, San Diego, CA, USA). Kaplan−Meier survival curve analysis was performed via the Mantel−Cox log-rank method for curve comparison analysis. All of the quantitative data shown in the graphs represent the mean ± standard error of the mean (SEM) of each group, and the statistical comparisons between the two groups were analyzed for significance via the nonparametric unpaired Mann−Whitney *U* test. Intergroup differences were assessed by one-way analysis of variance (ANOVA) followed by *post-hoc* analysis (Dunnett’s multiple comparisons test). Independent-sample *t*-tests, Chi-square tests, and Fisher’s exact probability tests were performed to compare patient characteristics between the two groups. A *p*-value < 0.05 was considered to indicate statistical significance.

## 3 Results

### 3.1 Successful establishment of an aGVHD mouse model

**Figure 1:**
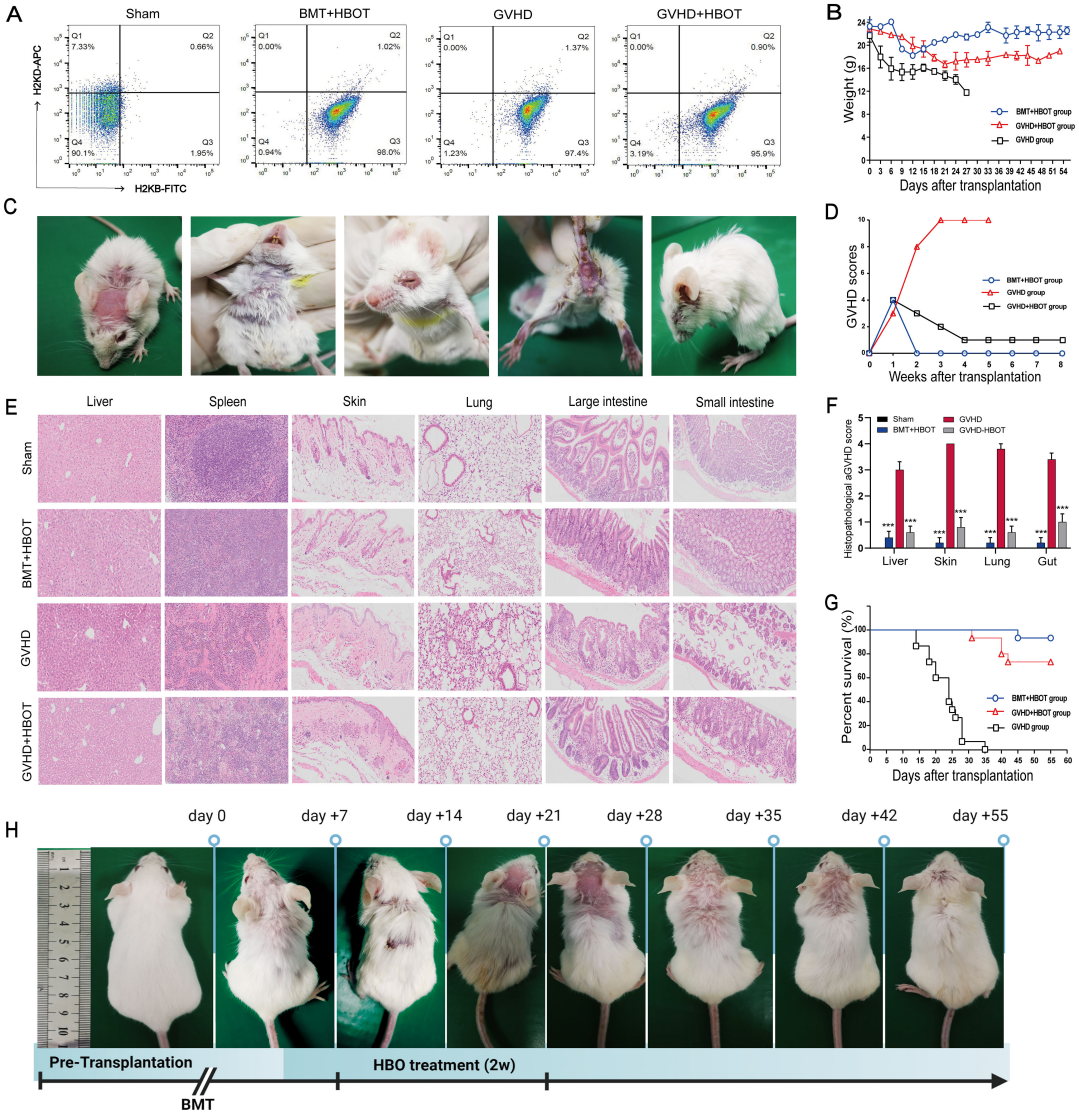
Successful establishment of an aGVHD mouse model and the alleviation of aGVHD by HBOT, with lower mortality and morbidity. **(A)** The percentage chimerism of H-2Kb donor cells in bone marrow was tested by flow cytometry on Day 10 posttransplantation. Staining revealed that 96.95% ± 1.05% of the cells were positive for a fluorescence signal from H-2Kb-FITC in the bone marrow cavity of the recipient mice, indicating that the donor bone marrow cells were completely chimeric. **(B)** The body weights of the mice were recorded once daily. **(C)** aGVHD group mice presented significantly increased symptoms of aGVHD on Day 28 posttransplantation, including a hunched appearance, ruffled fur, and alopecia combined with severe scurf on hair-free areas, diarrhea, and decreased activity. **(D)** The aGVHD scores of recipient mice were calculated according to the method of Cooke et al. **(E)** Hematoxylin and eosin (HE) staining of the liver, spleen, skin, lung, large intestine, and small intestine tissues from recipients. On Day 28 posttransplantation, the mice were killed, and pathological damage to the targeted organs was evaluated via HE staining. Compared with vehicle control recipients, aGVHD-HBOT recipients presented decreased pathological damage to targeted tissues. **(F)** Quantification of histological disease scores. **(G)** Survival time was monitored weekly, and the data were analyzed using Kaplan−Meier survival curves. **(H)** Changes in ruffled fur and skin defects after the administration of HBOT to aGVHD mice. ***P <.001.

On Day 10 after transplantation, the recipients achieved sustained, full donor chimerism (**Figure 1A**). All allogeneic transplanted mice began losing weight posttransplantation, reaching a nadir around Day 27, whereas mice receiving only bone marrow cells (BMT group) fully recovered by Day 21 without aGVHD-related symptoms (**Figure 1B**). In addition, mice that underwent allo-HSCT presented significant aGVHD symptoms, including a hunched appearance, ruffled fur, and alopecia with severe scurf on hair-free areas, diarrhea, and decreased activity, approximately 21 days posttransplant (**Figure 1C**). Clinical aGVHD scores remained consistently high until death due to severe rejection (**Figure 1D**). The pathophysiology was marked by infiltration and destruction of aGVHD target tissues (**Figures 1E, F**). Furthermore, allo-HSCT mice exhibited significantly reduced survival, with all recipients dying within 35 days posttransplantation due to severe aGVHD (**Figure 1G**). These results indicate that the aGVHD mouse model was successfully established.

### 3.2 HBOT alleviates aGVHD with reduced mortality and morbidity in a murine model

The effects of HBOT on the development of aGVHD were evaluated *in vivo* using an aGVHD mouse model. The effectiveness of HBOT was assessed based on weight loss, typical aGVHD symptoms, aGVHD scores, survival, and histological evaluation of aGVHD target tissues. All experimental cohorts experienced initial weight loss due to radiation toxicity, with the aGVHD group showing a rapid body weight reduction of up to − 40% around Day 27 posttransplantation until death. In contrast, the aGVHD+HBOT group exhibited an initial rapid weight loss within the first 3 weeks, followed by a gradual recovery, surpassing body weight levels of the BMT+HBOT cohort (**Figure 1B**).

The aGVHD group presented typical aGVHD-related symptoms within 20 days, whereas the aGVHD+HBOT group displayed only slightly ruffled fur on Day 40 posttransplantation (**Figure 1H**), with significantly lower aGVHD scores than the untreated group (**Figure 1D**). At 2 weeks posttransplantation, mice in the aGVHD+HBOT group showed markedly reduced aGVHD symptoms, including reduced weight loss (*p* < 0.01). Images were captured at 0, 7, 14, 21, 28, 35, 42, and 55 days after transplantation (**Figure 1H**). A significant difference in aGVHD scores was observed after Day 7 between the mice that received HBOT and those that did not. Similarly, histopathological analysis revealed reduced damage to multiple targeted organs, including the small intestine, large intestine, liver, spleen, and skin, in aGVHD+HBOT mice. The systemic manifestations of aGVHD were mildly associated with the amelioration of aGVHD pathology (**Figures 1E, F**).

As shown by the K-M curve, the HBOT recipients had significantly prolonged survival compared to untreated mice (log-rank, *p* = 0.005). All aGVHD mice eventually died within 1 month after transplantation (**Figure 1G**). In conclusion, HBOT mitigates weight loss, mortality, aGVHD symptoms, and targeted organ pathology associated with aGVHD. Compared with control aGVHD mice, HBOT-treated mice exhibited lower aGVHD scores and improved survival. The aGVHD mortality rate of vehicle-treated recipients was 100%.

### 3.3 HBOT reduces ROS production and serum cytokine levels

**Figure 2:**
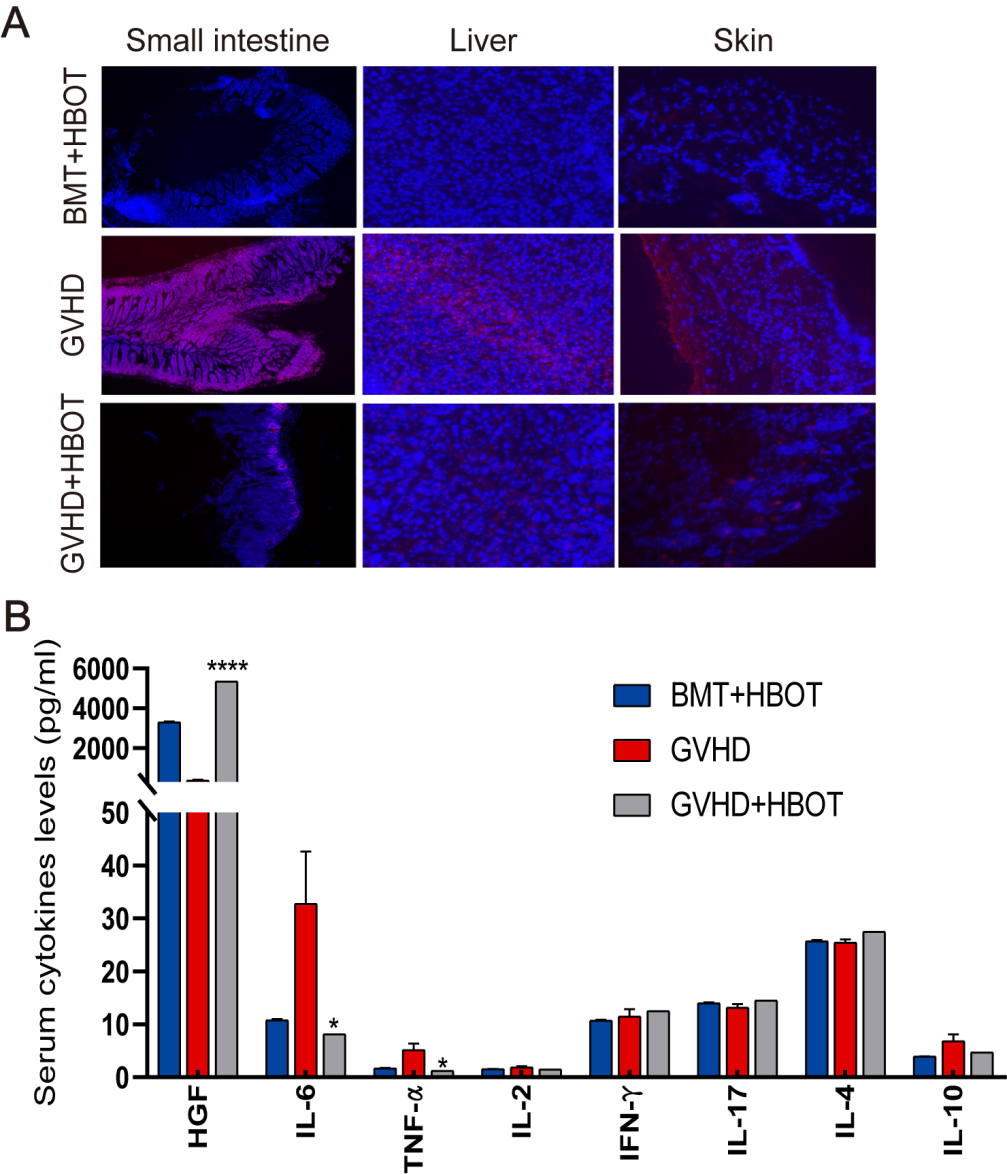
HBOT administration reduces ROS production and serum cytokine levels. **(A)** Representative fluorescence images of ROS staining using dihydroethidium (DHE) in the gut, skin, and liver tissues of recipients on Day 28 posttransplantation. **(B)** Terminal blood samples were collected from the inner canthus venous plexus of recipients 28 days after HSCT. The levels of various factors in terminal blood samples were measured using an 8-Plex Mouse Magnetic Luminex Assay. *P <.05, ****P <.0001.

To further understand the molecular mechanisms associated with the beneficial effects of HBOT, ROS levels in targeted organ tissues and serum cytokines were measured. Dihydroethidium (DHE) staining was performed on whole tissues, as their small size allows for DHE penetration. DHE staining revealed that aGVHD development in mice led to ROS accumulation in the gut, skin, and liver, triggering oxidative stress in these organs (**Figure 2A**). There was an increased number of DHE-positive cells in the aGVHD group. ROS accumulation peaked in aGVHD-related tissues, but was gradually cleared after HBOT. Immunofluorescence microscopy evaluation of mouse liver, skin, and small intestine showed a reduction in fluorescence intensity in the aGVHD-HBOT group, indicating that HBOT effectively reduced the aGVHD-related generation of ROS. These findings suggest that HBOT significantly alleviates aGVHD-induced damage by modulating the balance of oxidation and antioxidation. Serum analysis on Day 28 following allo-HSCT revealed that IL-6 and TNF-α proinflammatory cytokine levels were significantly lower (*p* < 0.05) in HBOT recipients than in aGVHD control recipients (**Figure 2B**). Hepatocyte growth factor (HGF), primarily expressed in the liver and used as a marker of aGVHD (24), was significantly higher in HBOT recipients than in untreated recipients (*p* < 0.0001). No significant differences were observed in other cytokines between the two groups (**Figure 2B**).

### 3.4 HBOT suppresses aGVHD by activating the hyperoxia-induced Nrf2/HO-1 pathway

**Figure 3:**
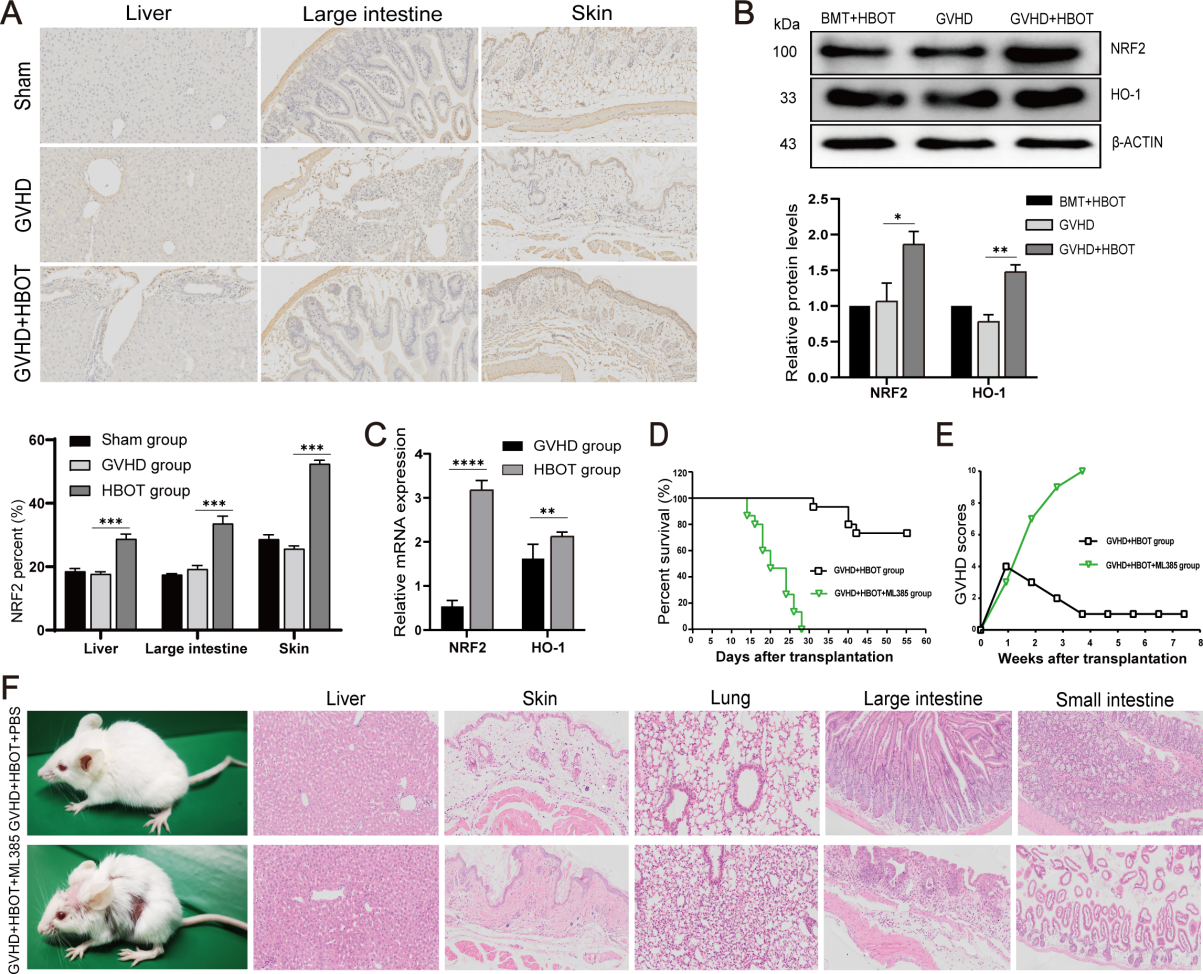
HBOT suppresses aGVHD by activating the hyperoxia-induced Nrf2/HO-1 pathway. **(A)** Nrf2 protein expression in the liver, large intestine, and skin was determined via immunohistochemical staining on Day 28 posttransplantation. **(B)** Nrf2 and HO-1 protein expression in the liver was determined using Western blot analysis on Day 28 posttransplantation. **(C)** Nrf2 and HO-1 mRNA levels in the liver were determined using real-time PCR. **(D)** Survival time was monitored weekly, and the data were analyzed via Kaplan−Meier survival curves. **(E)** The average aGVHD scores of the recipient mice were calculated according to the methods of Cooke et al. **(F)** Skin defects and HE staining in the aGVHD+HBOT+ML385 group and aGVHD+HBOT+PBS group. *P <.05, **P <.01, ***P <.001, ****P <.0001.

As Nrf2 regulates the expression of numerous genes that participate in the control of ROS, the present study verified whether Nrf2 plays a role in alleviating aGVHD after HBOT. Our findings indicated that inhibiting Nrf2 led to increased ROS levels in most tissues. Therefore, we first isolated aGVHD mouse livers and examined the expression of Nrf2 and HO-1. Western blot analysis and immunohistochemical staining revealed that the protein levels of Nrf2 and HO-1 were significantly lower in the liver, lung, skin, small intestine, and large intestine of aGVHD mice than in syngeneic control animals (**Figures 3A, B**), suggesting that Nrf2 and HO-1 suppression may be involved in the pathogenesis of aGVHD. Taken together, these findings suggested that the Nrf2/HO-1 signaling pathway plays a critical role in the development of aGVHD. The potential impact of HBOT on the Nrf2/HO-1 pathway, which is involved in aGVHD progression, was then evaluated. As shown in **Figures 3B, C**, both the Nrf2 and HO-1 mRNA and protein expression levels of Nrf2 and HO-1 in the liver were increased in recipients treated with HBOT without inducing inflammatory responses.

To further confirm that HBOT improves both aGVHD symptoms and histological lesions by activating the Nrf2/HO-1 pathway, an inhibitor of Nrf2 (ML385) was used to pretreat recipient aGVHD model mice. Previous studies have shown that ML385 decreases the protein levels of Nrf2 and HO-1, indicating that ML385 significantly inhibits the Nrf2/HO-1 signaling pathway (25). Compared with the PBS group, the aGVHD-HBOT-ML385 group exhibited shorter survival (**Figure 3D**) and more severe aGVHD symptoms (**Figures 3E, F**), as indicated by weight loss and skin defects. Moreover, HBOT did not alleviate aGVHD target organ damage in mice pretreated with ML385, as evidenced by increased inflammatory cell infiltration and more severe damage to the liver, lung, skin, small intestine, and colon (**Figure 3F**).

### 3.5 Efficacy and safety validation of HBOT in clinical practice

**Figure 4:**
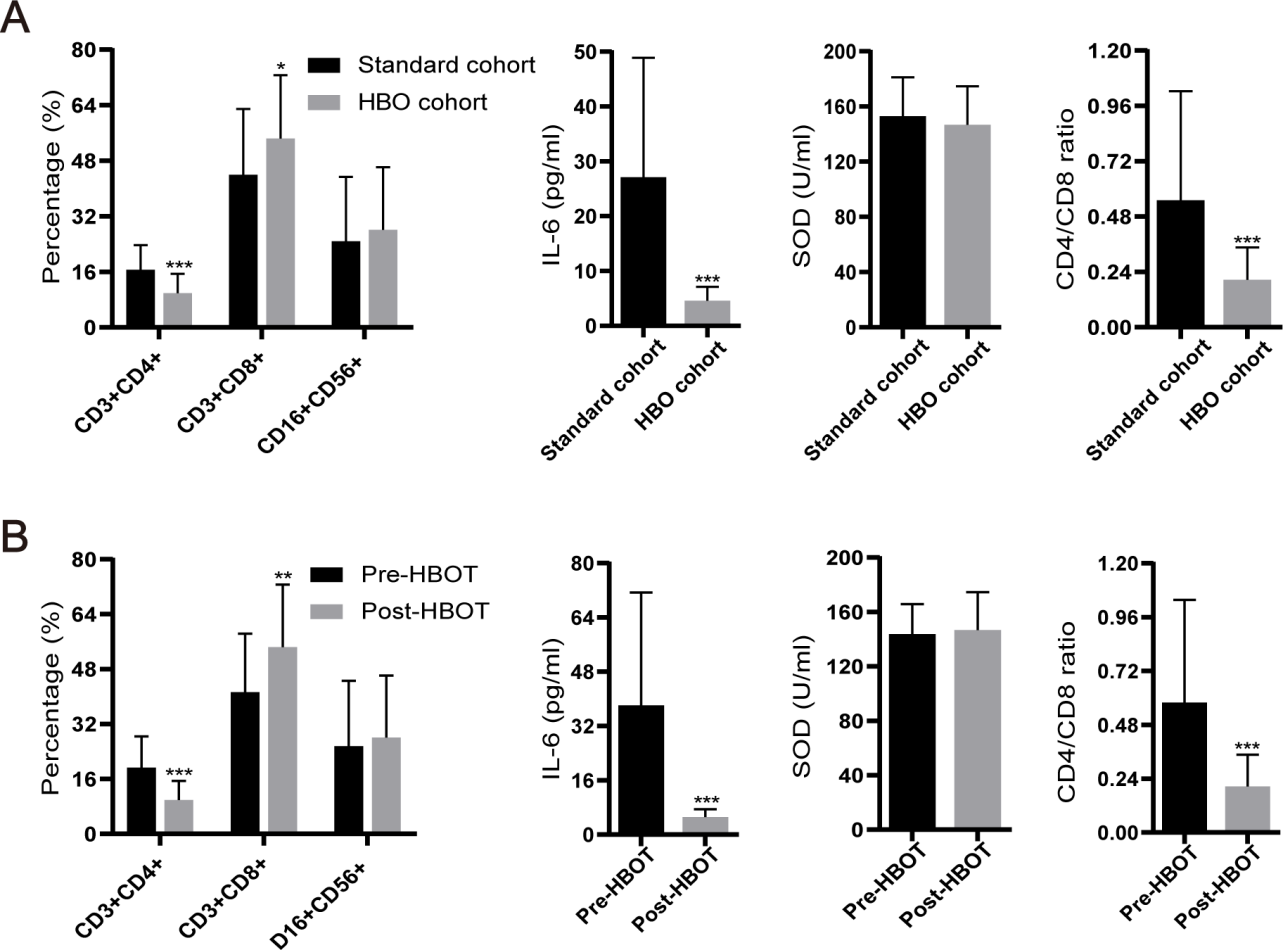
Levels of IL-6, superoxide dismutase (SOD), CD4+ T cells, and CD8+ T cells, as well as the CD4+ T/CD8+ T ratio, among clinical patients. **(A)** Comparison of various factors between the standard cohort and HBOT cohort. **(B)** Levels of various factors before and after HBOT. *P <.05, **P <.01, ***P <.001.

All 83 aGVHD patients predominantly received routine first-line treatment with glucocorticoids combined with CNIs. Thirty patients in the HBOT cohort completed the prescribed HBOT in addition to the standard first-line therapy. None of the patients interrupted or discontinued their treatment due to discomfort. The standard cohort included 53 patients who received only glucocorticoids combined with CNIs. **Table 1** summarizes the clinical characteristics of the 83 enrolled patients. Before treatment, 28 patients developed grades 1–3 aGVHD of the skin, 12 developed grades 1–3 aGVHD of the gut, and one patient developed grade 1 aGVHD of the liver. After continuous treatment with HBOT, only 15 patients developed grades 1–3 aGVHD of the skin, 10 developed grades 1–3 aGVHD of the intestinal tract, and none developed aGVHD involving the liver (**Table 2**). Regarding the general treatment response, although a direct comparison is not appropriate due to the significant difference in baseline aGVHD grades between the two cohorts (**Table 1**), a trend toward superior efficacy in managing grades 3–4 GVHD in the HBOT cohort was observed. Furthermore, more patients in the standard cohort experienced further exacerbation of aGVHD, particularly in the liver and intestines. There were no significant differences in treating grades 1–2 aGVHD in the two groups (**Table 3**). Among all aGVHD patients who underwent HBOT, the proportion of CD4+ T cells and serum IL-6 in the peripheral blood was significantly lower than that in the control, but this trend was reversed for CD8+ T cells (**Figure 4**).

**Table 1:** Patient demographics and baseline disease characteristics.

Clinical variable	Standard cohort No. (%)	HBOT cohort No. (%)	*p*-value
	*N* = 53	*N* = 30	
**Median (range) age (years)**	24 (8–65)	31 (8–60)	0.098
Age group (*n* (%))			
< 30 years	34 (64.2)	12 (40)	0.033
≥ 30 years	19 (35.8)	18 (60)	
Sex			
F	16 (30.2)	15 (50)	0.073
M	37 (69.8)	15 (50)	
Underlying diseases			
Acute lymphoblastic leukemia	15 (28.3)	7 (23.3)	0.455
Acute myeloid leukemia	22 (41.5)	19 (63.3)	
Severe aplastic anemia	4 (7.5)	2 (6.7)	
Myelodysplastic syndrome	4 (7.5)	0 (0)	
Lymphoma (HL, NHL)	4 (7.5)	1 (3.3)	
Others	4 (7.5)	1 (3.3)	
Number of transplants			
First HSCT	51 (96.2)	28 (93.3)	0.618
Second or more HSCT	2 (3.8)	2 (6.7)	
Time from transplant (month)^a^			
< 12	7 (13.2)	7 (23.3)	0.000
12–24	5 (9.4)	15 (50)	
≥ 24	41 (77.4)	8 (26.7)	
Donor type			
Related donor	51 (96.2)	29 (96.7)	0.624
Unrelated donor	2 (3.8)	1 (3.3)	
HLA matching			
5/10	26 (49.1)	16 (53.3)	0.934
> 5/10	24 (45.3)	12 (40)	
NA	3 (5.7)	2 (6.7)	
Sex mismatch			
M→M	19 (35.8)	9 (30)	0.403
M→F	13 (24.5)	10 (33.3)	
F→M	16 (30.2)	5 (16.7)	
F→F	4 (7.5)	5 (16.7)	
NA	1 (1.9)	1 (3.3)	
Graft type			
PB	47 (88.7)	25 (83.3)	0.515
BM+PB/CUB	6 (11.3)	5 (16.7)	
Conditioning regimen			
TBI/CY	5 (9.4)	1 (3.3)	0.522
mBU/CY	19 (35.8)	7 (23.3)	
IDA+BU/FLU	12 (22.6)	9 (30)	
FLU/CY	3 (5.7)	0 (0)	
VP16+BU/CY	9 (17)	7 (23.3)	
BU/FLU	5 (9.4)	4 (20)	
Time to engraftment (day)			
≤ 13	30 (56.6)	21 (70)	0.251
> 13	23 (43.4)	9 (30)	
Previous infectious episodes			
Yes	33 (62.3)	27 (90)	0.01
No	20 (37.7)	3 (10)	
Hemorrhagic cystitis^b^			
Yes	24 (45.3)	24 (80)	0.002
No	29 (54.7)	6 (20)	
Posttransplant day of aGVHD onset (day)			
≤ 30	38 (71.7)	21 (70)	0.972
31–60	6 (11.3)	5 (16.7)	
61–90	9 (17)	4 (13.3)	
Grade of aGVHD^c^			
Grade I	29 (54.7)	11 (36.7)	0.001
Grade II	7 (13.2)	15 (50)	
Grade III	17 (32.1)	4 (13.3)	
Organ-specific aGVHD severity stage			
Skin			
No aGVHD	9 (17)	2 (6.7)	0.066
1	21 (39.6)	10 (33.3)	
2	16 (30.2)	10 (33.3)	
3	7 (13.2)	8 (26.6)	
4	0 (0)	0 (0)	
Gut			
No aGVHD	33 (62.3)	18 (60)	0.732
1	7 (13.2)	8 (26.6)	
2	5 (9.4)	3 (10)	
3	4 (7.5)	1 (3.3)	
4	4 (7.5)	0 (0)	
Liver			
No aGVHD	44 (83)	29 (96.7)	0.064
1	5 (9.4)	1 (3.3)	
2	3 (5.7)	0 (0)	
3	1 (1.9)	0 (0)	
4	0 (0)	0 (0)	

*aGVHD*, acute-graft-versus host disease; *HBOT*, hyperbaric oxygen therapy; *UCB*, umbilical cord blood; *BM*, bone marrow; *PB*, peripheral blood stem cells; *F*, female; *M*, male; *HD*, Hodgkin lymphoma; *NHL*, nonHodgkin lymphoma; *HSCT*, hematopoietic stem cell transplantation; *HLA*, human leukocyte antigen; *TBI*, total body irradiation; *CY*, cyclophosphamide; *FLU*, fludarabin; *BU*, *busulfan*; *VP16*, etoposide; *mBU/FLU*, modified BU/FLU.

a The length of follow-up.

b Hemorrhagic cystitis at any time posttransplant.

c aGVHD presented before starting glucocorticoid or glucocorticoid+HBOT.

**Table 2:** Organ-specific aGVHD grade changes in patients treated with HBOT.

Organ-specific aGVHD grade	Pre-HBOT No. (%)	Post-HBOT No. (%)
No aGVHD	0 (0)	9 (30)
Skin		
1	10 (33.3)	9 (30)
2	10 (33.3)	4 (13.3)
3	8 (26.6)	2 (6.7)
Gut		
1	8 (26.6)	8 (26.7)
2	3 (10)	1 (3.3)
3	1 (3.3)	1 (3.3)
Liver		
1	1 (3.3)	0 (0)
2	0 (0)	0 (0)
3	0 (0)	0 (0)

*aGVHD*, acute graft-versus-host disease; *HBOT*, hyperbaric oxygen therapy.

**Table 3:** Comparison of overall treatment response and organ-specific responses between the standard and HBOT cohorts.

	Standard cohort No. (%)		HBOT cohort No. (%)	
	*N* = 53		*N* = 30	
	*Pretreatment*	*Posttreatment*	*Pretreatment*	*Posttreatment*
Grade of aGVHD				
Grade 0	0 (0)	34 (64.2)	0 (0)	21 (70)
Grade I	29 (54.7)	2 (3.8)	11 (36.7)	2 (6.7)
Grade II	7 (13.2)	3 (5.7)	15 (50)	5 (16.7)
Grade III	17 (32.1)	12 (22.6)	4 (13.3)	2 (6.7)
Grade IV	0 (0)	2 (3.8)	0 (0)	0 (0)
Organ-specific aGVHD severity stage				
Skin				
No aGVHD	9 (17)	48 (90.5)	2 (6.7)	15 (50)
1	21 (39.6)	2 (3.8)	10 (33.3)	9 (30)
2	16 (30.2)	2 (3.8)	10 (33.3)	4 (13.3)
3	7 (13.2)	0 (0)	8 (26.6)	2 (6.7)
4	0 (0)	1 (1.9)	0 (0)	0 (0)
Gut				
No aGVHD	33 (62.3)	41 (77.3)	18 (60)	20 (66.7)
1	7 (13.2)	0 (0)	8 (26.6)	8 (26.7)
2	5 (9.4)	8 (15.1)	3 (10)	1 (3.3)
3	4 (7.5)	3 (5.7)	1 (3.3)	1 (3.3)
4	4 (7.5)	1 (1.9)	0 (0)	0 (0)
Liver				
No aGVHD	44 (83)	43 (81)	29 (96.7)	30 (100)
1	5 (9.4)	3 (5.7)	1 (3.3)	0 (0)
2	3 (5.7)	3 (5.7)	0 (0)	0 (0)
3	1 (1.9)	4 (7.5)	0 (0)	0 (0)
4	0 (0)	0 (0)	0 (0)	0 (0)

*aGVHD*, acute graft-versus-host disease; *HBOT*, hyperbaric oxygen therapy.

## 4 Discussion

The present study demonstrated that HBOT-protected recipient mice who underwent allo-HSCT against aGVHD by activating the Nrf2/HO-1 pathway, inhibiting ROS production, and adjusting associated proinflammatory cytokines. These preliminary findings were validated in a clinical cohort. In our mouse model and clinical cohort, HBOT was safe without obvious side effects. Since HBOT apparatuses are available in most medical institutions, the application of HBOT is both feasible and economical. Thus, the present findings suggest that HBOT may be a potentially effective and safe option for aGVHD prophylaxis and treatment.

Previous studies have confirmed the efficacy and safety of HBOT in various diseases, such as aerobic poisoning, decompression sickness, gas embolism, and barotrauma. Marvin et al. revealed that HBOT improves nonhealing ulcers secondary to GVHD, chemotherapy, and/or radiation therapy-induced HC (10, 26). Our previous study demonstrated that HBOT was both effective and well-tolerated in patients with late-onset HC following allo-HSCT (27). The above aGVHD-associated symptoms are closely related to the activation of immune cells and the culmination of cytokine storms, which is similar to the process of aGVHD. Although these patients with aGVHD received first-line therapy, including glucocorticoids and CNIs, half still developed SR-aGVHD. Therefore, an urgent clinical need exists to explore innovative therapies for aGVHD.

In the present study, HBOT improved the symptoms and organ pathology of recipients with aGVHD and prolonged the survival of allo-recipients compared with the HBOT-free group. aGVHD is an immune-mediated disease resulting from the activation of donor lymphocytes by host antigen-presenting cells (APCs), followed by extensive clonal expansion and differentiation. The pathophysiology of aGVHD is not fully understood, and numerous studies have suggested that immunological mediators might play critical roles (28–30). HBOT also significantly reduces the production of ROS and proinflammatory cytokines. Oxidative stress also plays an unavoidable role in exacerbating GVHD (8, 31). Conditioning regimens, including high-dose chemotherapy and radiation, generally result in the accumulation of ROS in the organs of allo-HSCT patients (6, 7). Furthermore, the earliest pathophysiological events in aGVHD involve the infiltration of neutrophils, which serve as the initial immune response to organ injury. Neutrophils promote aGVHD through their activation and production of inflammatory mediators, such as IL-1β and ROS, which drive both innate and adaptive immune responses (4, 32–34). ROS and multiple cytokines are closely related to the severity and persistence of aGVHD. Qian et al. proposed that hydrogen therapy may be an effective and specific way to treat aGVHD (35). The present study revealed that the increased ROS levels in the targeted organs of aGVHD patients were markedly reduced after HBOT.

HBOT has been demonstrated to increase the partial pressure of oxygen in the blood, improve oxygen concentration, and accelerate the clearance of ROS and proinflammatory cytokines (36). In the present study, HBOT reduced the expression of IL-6 and TNF-α while upregulating the expression of HGF. These outcomes are in line with those of Al-Waili et al., who revealed that HBOT minimizes the proliferation of damaging lymphocytes while modulating the biology of inflammatory mediators and cytokines. Al-Walli et al. also reported that HBOT suppresses the production of proinflammatory cytokines and affects the liberation of TNF-α (37).

The present findings indicated that HBOT protects the host from aGVHD by activating the Nrf2 pathway. In the present study, the expression levels of Nrf2 and HO-1, a downstream antioxidant defense enzyme, were both upregulated in recipients given HBOT. Dhamodaran et al. confirmed that increased levels of Nrf2 transiently regulate the expression of angiogenic genes in the treatment of diabetic foot ulcers during HBOT (14). Meng et al. revealed that Nrf2 levels and the expression of its downstream targets, such as HO-1 and NQO-1, are upregulated during HBOT following traumatic brain injury (15). To the best of our knowledge, this is the first line of evidence indicating elevated levels of Nrf2 and HO-1 in recipients receiving HBOT. The present findings also demonstrated that treatment with an Nrf2 inhibitor reduced the protective effect of HBOT on aGVHD. In the present study, rescue experiments revealed that Nrf2 is indispensable for the protective effect of HBOT on aGVHD. Few studies have well documented that Nrf2 acts as a promising target for aGVHD therapy. Jisun et al. demonstrated that the CREB1−Nrf2 signaling pathway, along with improved GSH synthesis, is beneficial for the treatment of aGVHD (38). In addition, Jennifer et al. revealed that tumor-bearing allo-HSCT recipients of Nrf2^−/−^ donor T cells have prolonged OS as a result of a preserved graft-versus-leukemia (GVL) effect and reduced aGVHD activity, which was characterized as a novel therapeutic target to improve the outcomes of aGVHD (39).

In this study, the preliminary findings in the mouse model were validated in a clinical trial. The proportion of subjects with grades III and above decreased after HBOT, suggesting a promising clinical implication for HBOT in the management of grade III/IV aGVHD. Among all aGVHD patients who received HBOT, the levels of CD4+ T cells and serum IL-6 in the peripheral blood were significantly lower than those in the standard cohort. In contrast, the proportion of CD8+ T cells was markedly increased in aGVHD patients who received HBOT compared to the standard cohort. These findings suggested that CD8+ T cells are not influenced by HBOT and that the GVL effect is conserved. The GVL effect is primarily mediated by CD8+ T cells and NK cells, while CD4+ T cells mainly contribute to the development of aGVHD (40–43).

The present study had several limitations. First, the number of clinical samples was small. Future studies will employ a larger sample size to reduce the heterogeneity between individuals. Second, the present study focused only on the HBOT/Nrf2/HO-1 pathway in aGVHD. As HBOT may regulate multiple signaling pathways in different types of cells, it remains unclear whether other downstream signaling pathways are involved. Third, the mechanism by which HBOT reduces aGVHD while maintaining the GVL effect requires further validation. Finally, the present study applied HBOT as a therapeutic rather than a prophylactic measure in the human cohort. It is not feasible to administer HBOT within the first 7 days posttransplantation when patients are housed in sterile laminar flow wards following routine transplant procedures. In addition, it is unclear who will develop aGVHD or when it will develop, and preliminary findings on early HBOT after allo-HSCT are limited. Due to these factors and concerns regarding patient compliance and safety, HBOT was chosen as a therapeutic rather than prophylactic intervention. This experience may inform the future use of HBOT as a prophylactic strategy to improve efficacy in this population.

In summary, the present study demonstrated the safety and beneficial effects of HBOT in aGVHD using a murine model and validated these preclinical results in a clinical cohort. The findings indicated that preventive HBOT can improve aGVHD by activating the Nrf2/HO-1 signaling transduction pathway, suggesting that HBOT may be a feasible approach for aGVHD prevention and treatment. Additionally, the molecular and functional significance of the Nrf2/HO-1 pathway in protecting against aGVHD was elucidated. Activation of this pathway may suppress aGVHD by inhibiting the expression of downstream inflammatory genes and ROS. These findings may broaden the clinical indications of HBOT in the prophylaxis and treatment of aGVHD, particularly in reducing grade III/IV aGVHD. Future laboratory and clinical investigations are warranted to further confirm the promising application of HBOT in aGVHD.

## Data Availability

The original contributions presented in the study are included in the article/**Supplementary Material**. Further inquiries can be directed to the corresponding authors.
