# Supplementary material for: Preventive hyperbaric oxygen therapy improves acute graft-versus-host disease by activating the Nrf2/HO-1 pathway
Source: Front Immunol. 2025 Feb 27;16:1529176. doi: 10.3389/fimmu.2025.1529176 (PMC11903425; doi:10.3389/fimmu.2025.1529176)

Hyperbaric Oxygen Therapy Improve Acute Graft-Versus-Host Disease by Activating the Nrf2/HO-1 Pathway

HBOT can alleviate acute GVHD mice by decreasing the levels of ROS and proinflammatory cytokines, and upregulating the expression levels of Nrf2 and its downstream antioxidant enzymes HO-1

Acute GVHD can be alleviated by HBOT in mice model associated with a significantly prolonged OS and improved pathological injury.

The effect of HBO on alleviating acute GVHD target organ damage disappeared in mice pre-treatment with NRF2 inhibitor (ML385)

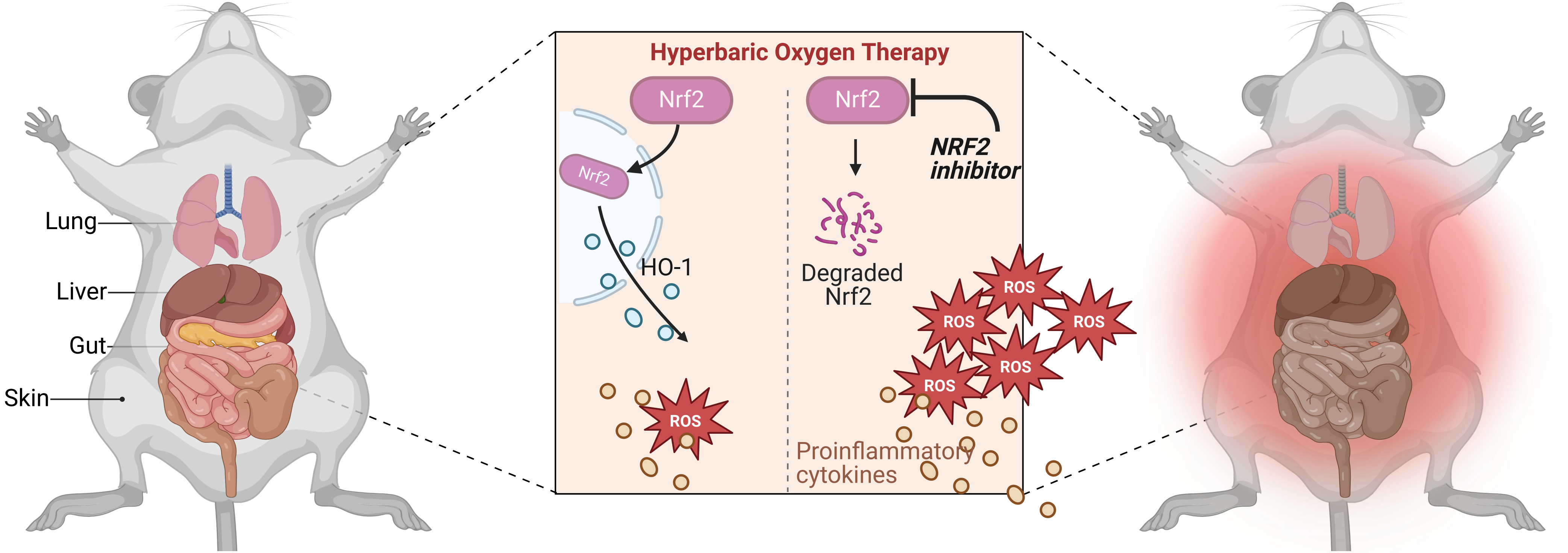

Supplement: Supplementary file 1 [file DataSheet1.pdf]
