# Supplementary material for: Preventive hyperbaric oxygen therapy improves acute graft-versus-host disease by activating the Nrf2/HO-1 pathway
Source: Front Immunol. 2025 Feb 27;16:1529176. doi: 10.3389/fimmu.2025.1529176 (PMC11903425; doi:10.3389/fimmu.2025.1529176)
Supplement: Supplementary file 2 [file DataSheet2.docx]

**SUPPLEMENTARY MATERIALS 1**


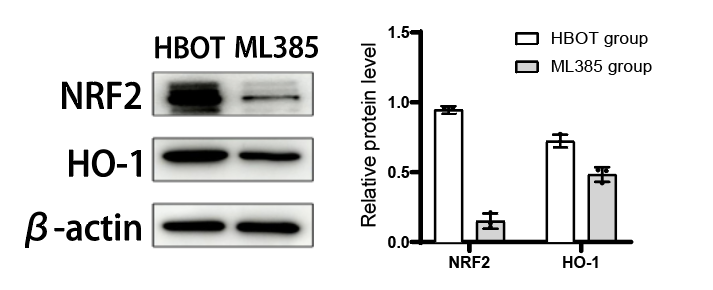


**Figure S1.** Effect of the ML385 inhibitor on NRF2 and HO-1 protein expression in HBOT-treated mice. Western blot analysis shows that treatment with ML385 in the HBOT group resulted in a significant reduction in both NRF2 and its downstream target, HO-1 protein levels.

**Table S1** Correlation between the number of splenocytes re-infused during GVHD model establishment and the manifestation of GVHD symptoms.

| **Group** | **TBI**  **(Gy)** | **Bone marrow cell**  **(×10^7^)** | | | **Spleen cell**  **(×10^7^)** | **Survival time**  **(d)** | **aGVHD symptom** |
| --- | --- | --- | --- | --- | --- | --- | --- |
| **TBI** | **7.5** | | **—** | **—** | | **＜7** | **No** |
| **BMT** | **7.5** | | **1** | **—** | | **>35** | **No** |
| **GVHD 1** | **7.5** | | **1** | **0.5** | | **>35** | **No** |
| **GVHD 2** | **7.5** | | **1** | **1** | | **>35** | **Mild** |
| **GVHD 3** | **7.5** | | **1** | **2** | | **＜35** | **Obvious** |
| **GVHD 4** | **7.5** | | **1** | **3** | | **＜14** | **Obvious** |

>35 considered as long-term survival; aGVHD, acute graft-versus-host disease; HSCT, hematopoietic stem cell transplantation; TBI, total body irradiation.
